# Clinical Outcomes of Switching from Prostaglandin Analogs to Omidenepag Isopropyl in Primary Open-Angle Glaucoma and Glaucoma Suspect Patients with Prostaglandin-Associated Periorbitopathy: A Prospective, Multicenter Study

**DOI:** 10.3390/ph19101636

**Published:** 2026-10-16

**Authors:** Jin Wook Jeoung, Jong Jin Jung, Hae-Young Lopilly Park, Joon Mo Kim, Chan Yun Kim, Michael S. Kook

**Affiliations:** 1 Department of Ophthalmology, Seoul National University Hospital, Seoul National University College of Medicine, 101 Daehak-ro, Jongno-gu, Seoul 03080, Republic of Korea; neuroprotect@gmail.com; 2 Department of Ophthalmology, Kim’s Eye Hospital, Konyang University College of Medicine, 136 Yeongsin-ro, Yeongdeungpo-gu, Seoul 07301, Republic of Korea; jiny0122@kimeye.com; 3 Department of Ophthalmology, Seoul St. Mary’s Hospital, College of Medicine, The Catholic University of Korea, 222 Banpo-daero, Seocho-gu, Seoul 06591, Republic of Korea; lopilly@catholic.ac.kr; 4 Department of Ophthalmology, Kangbuk Samsung Hospital, Sungkyunkwan University School of Medicine, 29 Saemunan-ro, Jongno-gu, Seoul 03181, Republic of Korea; kjoonmo1@gmail.com; 5 Department of Ophthalmology, Severance Hospital, Yonsei University College of Medicine, 50-1 Yonsei-ro, Seodaemun-gu, Seoul 03722, Republic of Korea; kcyeye@yuhs.ac; 6 Department of Ophthalmology, Asan Medical Center, University of Ulsan College of Medicine, 88 Olympic-ro 43-gil, Songpa-gu, Seoul 05505, Republic of Korea

**Keywords:** glaucoma, omidenepag isopropyl, prostaglandin analogs, prostaglandin-associated periorbitopathy

## Abstract

**Background/Objectives:** Prostaglandin-associated periorbitopathy (PAP) is a common treatment-related burden of prostaglandin analog (PGA) therapy that may compromise long-term glaucoma management. This study evaluated improvement in PAP and changes in intraocular pressure (IOP) after switching from PGAs to omidenepag isopropyl. **Methods:** This prospective, multicenter study was conducted in Korea over 24 weeks. Adults diagnosed with PAP who had received PGA eye drops for ≥3 months for primary open-angle glaucoma, normal-tension glaucoma, or glaucoma suspect were switched to omidenepag isopropyl. PAP manifestations were evaluated using investigator assessments, independent assessments, and patient-reported outcome measures. Changes in IOP and safety outcomes were also assessed. **Results:** A total of 96 patients were included in the final analysis. At Week 24, improvement in deepening of the upper eyelid sulcus was observed in 78.16% of patients. Improvements were also observed in flattening of the lower eyelid bag, upper eyelid ptosis, ciliary hypertrichosis, and periorbital hyperpigmentation. Mean IOP was significantly reduced from baseline (−2.14 mmHg), with 42.71% and 27.08% of patients achieving IOP reductions of ≥15% and ≥20%, respectively. Findings were consistent overall across institutional, independent, and subjective assessments. Most adverse events were mild, and no serious adverse events were reported. **Conclusions:** PAP manifestations improved following transition from PGA therapy to omidenepag isopropyl, while IOP control and favorable tolerability were maintained in glaucoma or glaucoma suspect patients with PAP. These findings may support the clinical feasibility of switching from PGAs to omidenepag isopropyl in patients with PAP.

## 1. Introduction

Glaucoma is a chronic progressive optic nerve disorder that can result in visual field loss and eventual irreversible blindness [1,2,3]. The global prevalence of glaucoma is estimated to reach 3.5% among individuals aged ≥40 years by 2060, with primary open-angle glaucoma (POAG) being the most common subtype. Glaucoma is estimated to affect 186.6 million people by 2060, predominantly in Asia and Africa [4,5]. Asia accounts for nearly 60% of all global glaucoma cases and contributes to about 53.4% of POAG cases worldwide [5]. Although there are various risk factors for glaucoma, elevated intraocular pressure (IOP) is the primary and only modifiable risk factor for the development and progression of POAG and ocular hypertension (OHT) [6,7,8].

Treatment aimed at lowering elevated IOP remains the primary approach for glaucoma management. Numerous clinical trials have demonstrated that reducing the elevated IOP decreases the risk of glaucoma development and progression [6,7,9]. Prostaglandin F receptor agonists (FP agonists), commonly known as prostaglandin analogs (PGAs), are the most widely prescribed first-line treatment for POAG and OHT due to their once-daily dosing and effective IOP reduction [10,11,12,13,14,15]. FP agonists effectively reduce IOP by stimulating aqueous humor outflow from the uveoscleral outflow tract [3,16,17]. However, many patients do not respond to FP agonists [18,19], and their use is associated with ocular cosmetic side effects collectively referred to as prostaglandin-associated periorbitopathy (PAP) [3,14,20,21,22]. PAP is associated with several clinical and cosmetic changes, including iris and periorbital skin pigmentation, eyelash elongation and thickening, and deepening of the upper eyelid sulcus (DUES) [14,20,21,22]. Furthermore, PAP can complicate patient management by making IOP measurement more challenging and increasing the risk of complications during surgery [23,24]. Therefore, the burden of PAP highlights an important unmet need for effective alternative treatment options in glaucoma management.

Omidenepag isopropyl is a novel, selective prostanoid E2 (EP2) receptor agonist approved for the treatment of POAG and OHT in the United States, Japan, and several Asian countries [25,26,27]. Omidenepag isopropyl lowers IOP by increasing aqueous humor drainage through both the conventional (trabecular) and uveoscleral outflow pathways [28,29]. Several studies have demonstrated the effectiveness of omidenepag isopropyl in lowering IOP in patients who are non/low-responders to latanoprost [30,31] and have shown its non-inferiority to latanoprost and timolol [30,32,33,34]. Although clinical trials and real-world evidence (RWE) have demonstrated improvements in PAP with omidenepag isopropyl, these studies were of shorter duration, with relatively smaller sample sizes, and fewer clinical outcomes [30,35,36,37]. This prospective, multicenter study aimed to evaluate the efficacy and safety of omidenepag isopropyl ophthalmic solution 0.002% in patients with glaucoma or who were glaucoma suspects diagnosed with PAP after switching from prostaglandin analogs for ≥3 months.

## 2. Results

### 2.1. Patient Disposition and Baseline Characteristics

**Figure 1:**
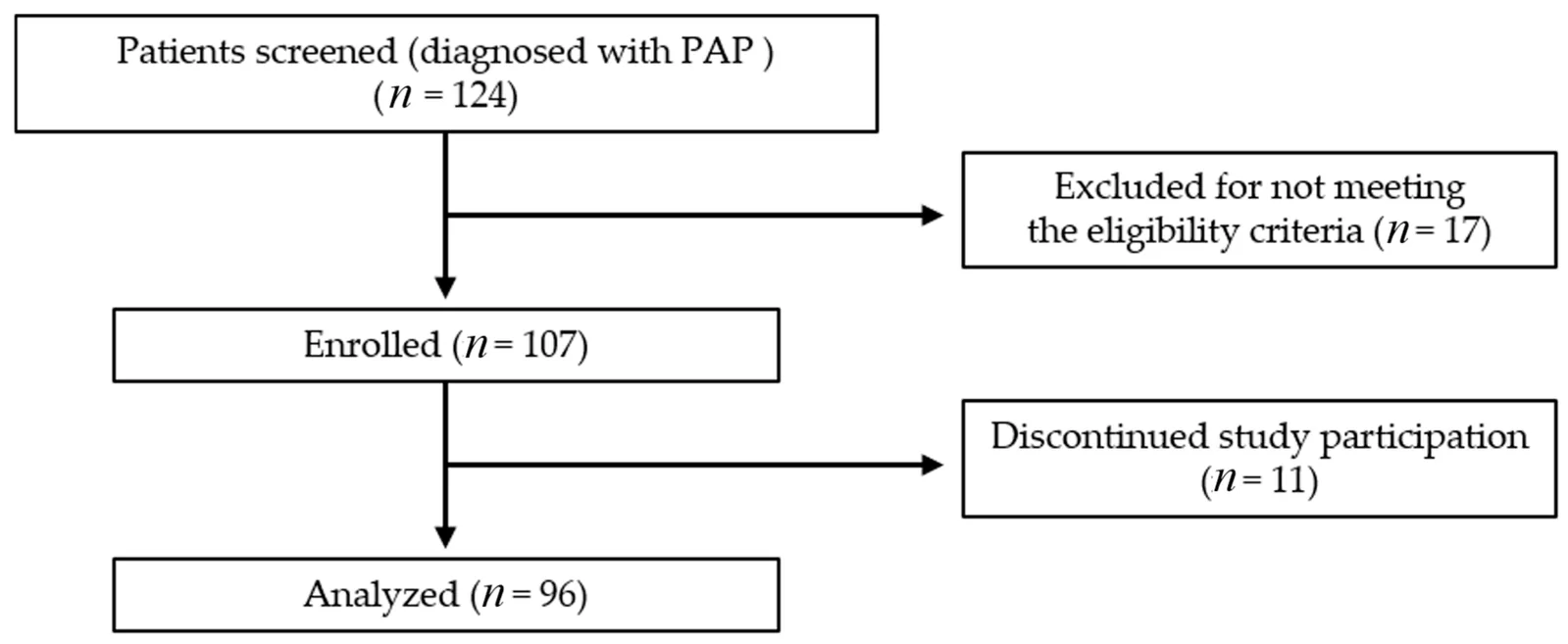
Patient disposition. PAP, prostaglandin-associated periorbitopathy.

Among the 124 patients screened, 17 did not meet the eligibility criteria and were excluded from the study. A total of 107 patients were enrolled in the study. Of these patients, 11 patients who discontinued and withdrew from the study were excluded from the analysis set due to withdrawal of consent (*n* = 5), use of a prohibited medication (*n* = 1), violation of eligibility criteria (*n* = 1), investigator decision (*n* = 2), and adverse events (*n* = 2). As a result, 96 patients were included in the final analysis (Figure 1).

The baseline demographic and clinical characteristics of the study population are summarized in Table 1. The mean age of patients in the analysis was 63.17 ± 10.46 years, and 57.29% were females. Regarding glaucoma diagnosis, 44.79% of patients had normal-tension glaucoma (NTG), 41.67% were glaucoma suspects, and 13.54% had POAG. Prior to switching to omidenepag isopropyl, PGA therapy included latanoprost (39.58%), bimatoprost (20.83%), tafluprost (20.83%), and travoprost (18.75%). A total of 64 patients (66.67%) had a history of surgery or medical history within the previous 6 months. The most frequently reported comorbidities were metabolic and nutritional disorders (29.65%), primarily including hyperlipidaemia (12.79%) and diabetes mellitus (8.72%), followed by vascular disorders (19.77%), mainly hypertension (16.28%), and eye disorders (13.37%), most commonly dry eye (6.40%) and cataract (3.49%).

Baseline PAP characteristics are summarized in Table 2. DUES and periorbital hyperpigmentation were evaluated using a 5-point grading scale (grades 0–4), whereas upper eyelid ptosis was assessed using a 3-point severity scale (mild, moderate, or severe). Flattening of lower eyelid bag (FLEB) and ciliary hypertrichosis were evaluated based on their presence (Yes) or absence (NO).

### 2.2. Primary Endpoint

At Week 24, 78.16% (*n* = 68/87) of patients showed improvement in DUES (excluding patients with a grade 0 DUES at baseline), as assessed by institutional investigators. This observed improvement rate was numerically higher than the historical improvement rate of 50% derived from a previous study [38] (Table 3).

### 2.3. Secondary Endpoints

Based on assessments by institutional investigators, the improvement rate for flattening of lower eyelid bag (FLEB) was 30.36% at Week 24 compared with baseline (excluding patients with no FLEB at baseline), which was higher than the historical improvement rate of 16.67%. At Week 24, compared with baseline, improvements were observed in 43.86% of patients with upper eyelid ptosis (excluding those with mild upper eyelid ptosis at baseline) and in 49.32% of patients with ciliary hypertrichosis (excluding those with no ciliary hypertrichosis at baseline). Both rates were higher than the historical improvement rate of 8.33%. The majority of patients showed improvements in periorbital hyperpigmentation (75.27%; excluding patients with a grade 0 periorbital hyperpigmentation at baseline), exceeding the historical improvement rate of 41.67% [38] (Table 3).

**Figure 2:**
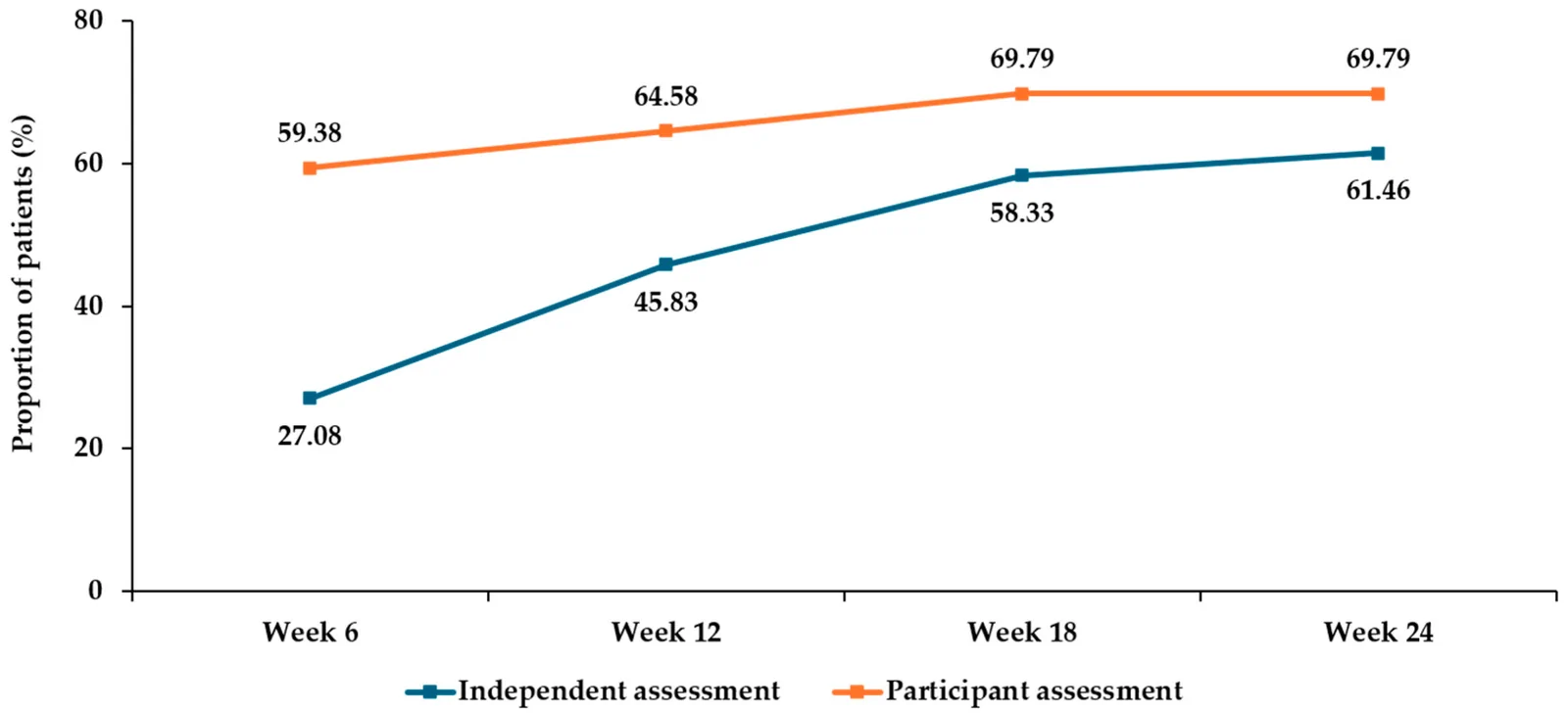
Evaluation of changes in PAP over time, as evaluated by independent evaluators and patients. PAP, prostaglandin-associated periorbitopathy.

Supporting these findings, improvement rates assessed by three independent evaluators using facial photographs increased progressively over time, from 27.08% at Week 6 to 45.83% at Week 12, 58.33% at Week 18, and 61.46% at Week 24. Similarly, subjective assessments reported by patients demonstrated a consistent trend toward PAP improvement across successive time points: 59.38% at Week 6, 64.58% at Week 12, and 69.79% at both Week 18 and Week 24, respectively (Figure 2).

**Figure 3:**
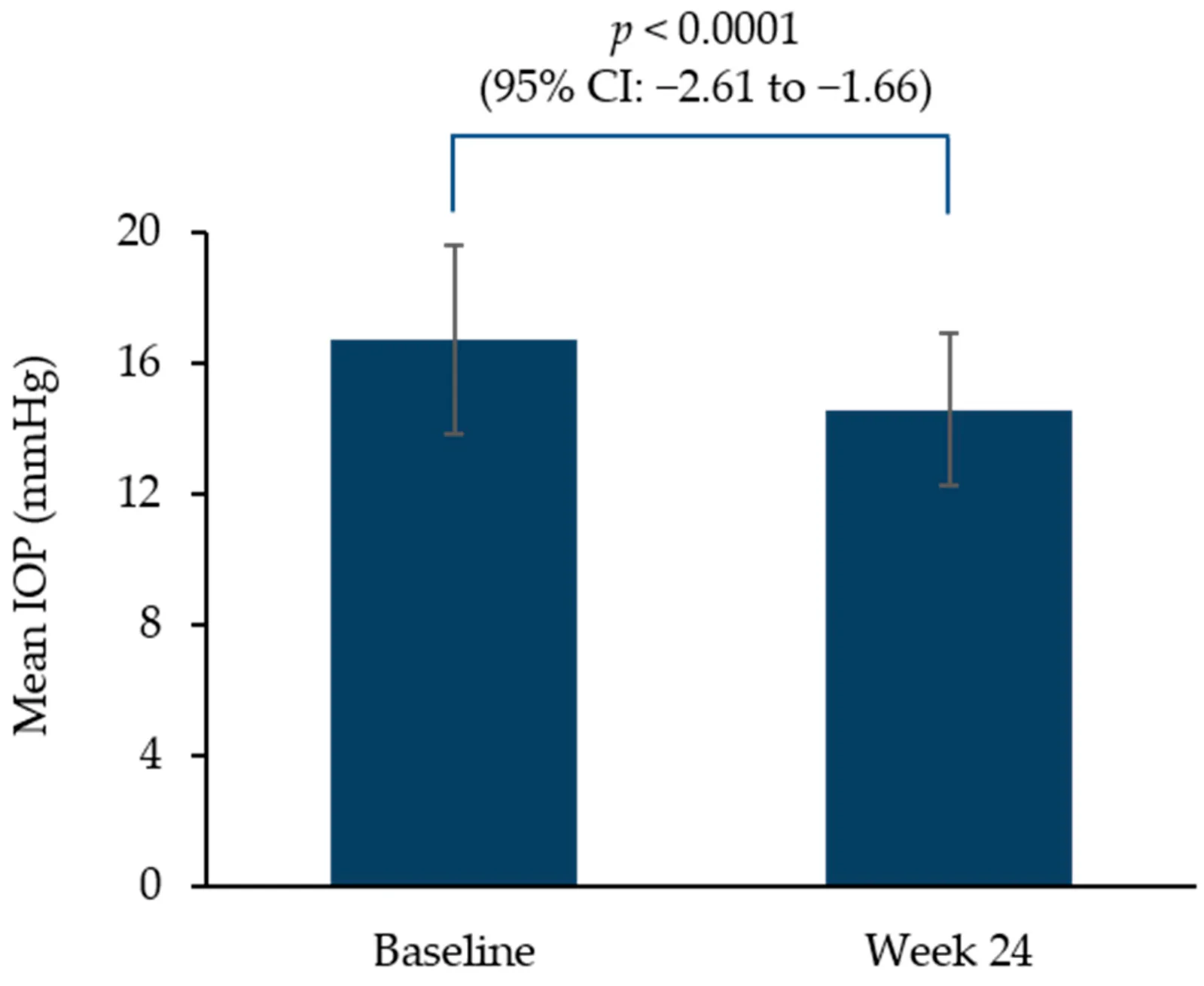
Change in mean IOP from baseline to Week 24. Error bars represent standard deviations. IOP, intraocular pressure.

The mean IOP was significantly reduced at Week 24 (14.57 ± 2.34 mmHg) compared with baseline (16.71 ± 2.86 mmHg), with a mean difference of −2.14 ± 2.33 mmHg (*p* < 0.0001; 95% CI: −2.61 to −1.66) (Figure 3). A 12.8% reduction in mean IOP was observed at Week 24 compared with baseline. Compared with baseline, 41 patients (42.71%) achieved an IOP reduction of ≥15% and 26 patients (27.08%) achieved an IOP reduction of ≥20% at Week 24. Mean IOP at screening while patients were still receiving PGA therapy was 14.56 ± 3.00 mmHg and remained at a similar level at Week 24 after switching to omidenepag isopropyl (Supplementary Table S1).

### 2.4. Safety

Among the 107 patients in the safety population during the treatment period, 22 patients (20.56%) reported 33 AEs, of which 32 AEs (96.97%) were mild, and 1 AE (3.03%) was moderate. Among these, 25 events in 14 patients (13.08%) were considered drug-related AEs. No serious AEs or serious drug-related AEs were reported. Unexpected adverse drug reactions (ADR) were reported in six cases involving two patients (1.87%), including myopia, chronic gastritis, hepatic calcification, hepatic haematoma, benign neoplasm, and liver hernia. The adverse events leading to treatment discontinuation were eye irritation and eye swelling in one patient and myopia in another patient. The most frequently reported AEs were eye-related (23 cases; 69.70%) and hepatobiliary disorders (3 cases; 9.09%) based on system organ class. Ocular hyperemia was the most frequent eye-related AE, with five cases (15.15%), followed by eye pain and reduced visual acuity, each reported in three cases (9.09%) (Table 4).

At Week 24, among the 96 patients evaluated, 94 patients (97.92%) had no macular edema (Grade 0), while 2 patients (2.08%) had Grade 1 macular edema. No cases of Grade 2 or higher macular edema were observed. For cystoid macular edema (CME), 94 patients (97.92%) showed no evidence of CME, whereas CME I and CME II were each reported in 1 patient (1.04%). No cases of CME III or CME IV were observed.

## 3. Discussion

This prospective, multicenter study extends previous evidence by providing a comprehensive evaluation of PAP changes in a relatively large cohort using complementary institutional, independent, and subjective assessments. Improvements were observed in multiple PAP manifestations after switching from PGA eye drops to omidenepag isopropyl ophthalmic solution, with consistent findings across all three assessment approaches, while effective IOP control was maintained.

Among the evaluated PAP manifestations, DUES and periorbital hyperpigmentation showed the highest improvement rates, with more than three-quarters of patients showing investigator-assessed improvement by Week 24. Improvement in both manifestations was evident as early as Week 6 and at subsequent visits through Week 24 (Supplementary Table S2). We also observed improvements in FLEB, upper eyelid ptosis, and ciliary hypertrichosis, all of which demonstrated progressive improvement from Week 6 through Week 18 (Supplementary Table S2). These findings are consistent with prior reports indicating that switching from PGF2α analogs to omidenepag isopropyl may lead to partial reversal of PAP manifestations [38,39]. Across all five PAP manifestations evaluated, the observed improvement rates were numerically higher than the historical improvement rates reported in the previous study [38]. However, given the exploratory nature of this study, comparisons with these historical improvement rates should be interpreted cautiously because of the limited sample size of the historical cohort and the uncertainty surrounding the reported estimates. Further studies with larger sample sizes are needed to confirm these findings. Our DUES findings are consistent with those of a prospective Japanese study, which reported objective improvement in DUES in 57% of patients within the first month and in 76% after 7 months of follow-up [36,37]. DUES has been reported to be associated with PGF2α-induced fat atrophy, primarily mediated through inhibition of adipocyte differentiation and adipogenesis [40,41]. In contrast to FP agonists, omidenepag isopropyl, a selective EP2 receptor agonist, does not affect adipocyte differentiation [42]. Therefore, this mechanistic difference may explain the observed improvement of DUES following transition from PGA therapy to omidenepag isopropyl in our study. In addition, the likelihood of developing hyperpigmentation is much lower with omidenepag isopropyl compared with PGAs, which may reduce the risk of further PAP-related hyperpigmentation changes [33,34].

Previous studies in Korean patients have identified DUES as the most prevalent manifestation of PAP, followed by periorbital hyperpigmentation [43]. As a deep upper eyelid sulcus is not a typical periocular feature in many Asian individuals, the development of DUES may be particularly noticeable and cosmetically concerning in this population [43,44]. Accordingly, effective management of DUES and periorbital hyperpigmentation represents an important unmet need among Asian patients receiving PGA therapy. Our findings suggest that these manifestations may be at least partially reversible following treatment modification, highlighting the potential clinical utility of omidenepag isopropyl in addressing this unmet need.

In contrast, FLEB and upper eyelid ptosis demonstrated comparatively lower rates of recovery. Previous studies have similarly reported limited alleviation of certain PAP manifestations, particularly FLEB, upper eyelid ptosis, and ciliary hypertrichosis, after discontinuation of PGAs [21,38]. These findings suggest that not all PAP manifestations are equally reversible and that certain PAP-related changes may persist once established despite treatment modification. Therefore, prevention of PAP may be preferable to attempting to alleviate established PAP, underscoring the importance of considering PAP risk when selecting initial glaucoma therapy.

In addition, independent and patient-reported assessments showed trends similar to those observed in the institutional assessment, with improvements observed from Week 6 through Week 24. The observed results were consistent across the institutional, independent, and patient-reported assessments, reinforcing the robustness of the observed improvements in PAP manifestations. Institutional assessment reflects clinical evaluation by investigators who were directly involved in patient care, ensuring relevance to routine practice. On the other hand, independent assessment conducted by external evaluators using standardized photographic documentation minimizes observer bias and enhances objectivity of the findings. Additionally, subjective assessment captures patient-reported outcome measures of periocular changes, providing valuable insights into cosmetic and functional impacts of therapy from patients’ perspectives.

As PAP may influence patients’ perception of treatment and potentially affect treatment adherence, avoiding PAP is an important consideration in long-term glaucoma therapy [21,36]. In the present study, omidenepag isopropyl demonstrated favorable tolerability, and most patients (89.72%) remained on treatment throughout the 6-month study period. Combined with the observed improvements in PAP manifestations, these findings suggest that omidenepag isopropyl can be a valuable long-term treatment option for patients with PAP.

Beyond its beneficial effects on PAP manifestations, omidenepag isopropyl also provided clinically meaningful IOP reduction. In the present study, IOP reduction was observed as early as Week 6 after switching to omidenepag isopropyl, suggesting an early onset. At week 24, omidenepag isopropyl achieved a mean IOP reduction of 12.8% from baseline. Notably, 42.71% and 27.08% of patients achieved IOP reductions of ≥15% and ≥20%, respectively. Despite the relatively low baseline IOP of the study population after the washout period (16.71 ± 2.86 mmHg), the observed magnitude of IOP reduction can be considered clinically meaningful in real-world clinical practice. Previous reports have suggested that the IOP-lowering effect of omidenepag isopropyl may be influenced by baseline IOP, with a lower baseline IOP generally associated with smaller reductions [28]. Therefore, this finding is particularly noteworthy given that 44.79% of the study population had normal-tension glaucoma.

These results are also in line with findings from a phase III clinical trial conducted in Japan, which demonstrated the non-inferiority of omidenepag isopropyl to latanoprost, one of the most widely prescribed FP agonists, in lowering IOP [32,33,34,45]. Previous studies have reported IOP reductions ranging from 20% to 35% with omidenepag isopropyl and efficacy comparable to that of PGAs when used as a first-line therapy [28]. Previous studies have shown that the IOP-lowering efficacy of omidenepag isopropyl can be sustained for up to 5 years [33,46].

Overall, omidenepag isopropyl 0.002% ophthalmic solution was well tolerated, with no serious AEs or serious drug-related AEs reported. Twenty-two patients experienced AEs, of which almost all were reported as mild. In this study, eye disorders were the most frequently reported AEs, followed by hepatobiliary disorders.

This study is strengthened by its relatively large, multicenter cohort of 96 patients compared with previously published studies evaluating PAP outcomes following switching to omidenepag isopropyl, which generally included fewer than 30 patients [36,38,39]. The larger sample size enabled a broader assessment of changes in five different PAP features and reduced the uncertainty associated with estimates from small sample sizes. Furthermore, the multicenter design extends the evidence from previous small-scale studies and provides a broader characterization of PAP changes in a more diverse clinical population. Another strength of this study is the standardized evaluation of five distinct PAP manifestations using predefined grading or categorical scales, with improvement determined according to prespecified changes from baseline. Moreover, PAP changes were evaluated using three complementary assessment approaches: institutional assessment by participating investigators, independent assessment by three external evaluators based on standardized photographs, and patient-reported assessment. The use of these approaches improved the objectivity of the evaluation. In addition, the consistency of findings across the three assessment methods further strengthens the observed results.

Our study has several limitations. First, the study is restricted by its single-arm design and the lack of a comparator group. To accurately assess the natural course of PAP and its changes over time, direct comparisons with a placebo group or a comparator are necessary. Although baseline assessments were performed after completion of a 4-week washout period and PAP outcomes were evaluated following the switch to omidenepag isopropyl, inclusion of a comparator group undergoing PGA discontinuation alone would have provided greater insight into the relative contributions of PGA withdrawal and treatment switching. Future comparative studies including a comparator group receiving continued PGA treatment or PGA discontinuation alone may further clarify the changes in PAP following the switch to omidenepag isopropyl. However, the aim of this study was to analyze the outcomes and trends associated with the use of omidenepag isopropyl from baseline to 24 weeks. Second, the 24-week observation period may not be sufficient to evaluate complete reversal of PAP. Previous studies have reported that full recovery from PAP may take several months to years, highlighting the need for long-term follow-up [36,47]. Third, upper eyelid ptosis was not assessed using a standardized or validated ptosis severity grading method. Instead, ptosis severity was evaluated using an individualized approach that referenced MRD2 measurements to account for inter-individual anatomical variability. Although this approach was intended to provide a more individualized assessment, future studies incorporating standardized ptosis grading methods would be valuable to further validate the observed findings. Fourth, glaucoma severity was not systematically assessed or classified, as this was not required by the study protocol. Therefore, the influence of disease severity on the observed outcomes could not be evaluated. Collection of glaucoma severity data and analyses according to disease severity may provide additional insight into outcomes following the switch to omidenepag isopropyl. Finally, concomitant systemic beta-blocker use and nocturnal hypotension were not specifically evaluated in this study. As both factors may influence glaucoma-related outcomes, including IOP measurements, their potential impact should be considered when interpreting the study findings.

## 4. Materials and Methods

### 4.1. Study Design

This single-arm, prospective, multicenter, open-label, phase IV clinical trial was conducted at six ophthalmic centers in Korea between February 2022 and March 2025 (ClinicalTrials.gov identifier: NCT05279716). The study duration was 24 weeks after a 4-week wash-out period (±1 week). Patients underwent clinical assessments at screening, baseline, Week 6 (±7 days), Week 12 (±7 days), Week 18 (±7 days), and Week 24 (±7 days) to collect data on safety and efficacy. The study was approved by the Institutional Review Board at each institution and was conducted in accordance with the ethical principles of the Declaration of Helsinki, Korean Good Clinical Practice and related regulations. Written informed consent was obtained from all patients.

### 4.2. Patients

Eligible patients were adults aged ≥19 and <80 years; diagnosed with NTG, POAG, glaucoma suspect, or OHT; received monotherapy with PGA eye drops for ≥3 months prior to consent; diagnosed with PAP associated with the use of PGA eye drops, with at least one eye showing one or more of the following: DUES, FLEB, upper eyelid ptosis, ciliary hypertrichosis, or periorbital hyperpigmentation.

The key exclusion criteria included glaucoma due to secondary causes such as pseudo-exfoliative glaucoma or pigment dispersion syndrome; IOP > 34 mmHg; severe visual field defects (mean deviation worse than −20 dB); history of ocular surgery including corneal refractive surgery (e.g., laser-assisted in situ keratomileusis or laser epithelial keratomileusis), or intraocular surgery within 6 months prior to consent; severe dry eye requiring or receiving treatment or presence of ocular allergy, infection, or inflammatory disease; use of systemic or topical ophthalmic steroids (excluding topical dermatologic use); the use of glaucoma medications other than PGAs; pregnant or breastfeeding women; known hypersensitivity to topical anesthetics, fluorescein, or the investigational product; subjects requiring contact lens use during the study period; eyes with pseudophakia or aphakia; history of trauma, surgery, or thyroid orbitopathy affecting the periocular area that could interfere with PAP evaluation.

### 4.3. Study Treatment and Assessments

Patients applied one drop of omidenepag isopropyl (EYBELIS ophthalmic solution 0.002%, Santen Pharmaceutical Co., Ltd., Osaka, Japan) to the affected eye(s) once daily for 24 weeks. The use of eye drops was permitted for patients with mild to moderate dry eye. Patients were given eye drop diaries to record the number of instillations of the study medication and dry eye medication. Patients who met the eligibility criteria underwent a washout period of 4 weeks (±1 week) from the date of screening. In addition, the following data were collected at screening: demographic information, ophthalmic or other medical conditions, visual acuity (uncorrected and best-corrected), IOP, PAP, and prior and concomitant medications.

For efficacy assessments, a predefined study eye was selected for each patient. When only one eye met the eligibility criteria, that eye was selected as the study eye. When both eyes met the eligibility criteria, the eye with more clinically severe PAP was selected as the study eye by the principal investigator at the time of enrollment. All efficacy assessments were performed using the selected study eye.

Facial photographs, including the eyebrows and lower eyelids, were taken before initiating omidenepag isopropyl eye drops and at subsequent study visits using the same digital camera under consistent indoor lighting conditions. The initial photograph served as the baseline, and all subsequent images were captured using the same method.

The primary endpoint was the proportion of patients with improvement in DUES at Week 24 compared with baseline, as assessed by the institutional investigators. DUES was graded on a 5-point scale ranging from 0–4: grade 0 = none; grade 1 = trace, barely visible; grade 2 = mild; grade 3 = moderate, easily detected; grade 4 = severe.

The secondary endpoints included the proportion of patients with improvement in FLEB, upper eyelid ptosis, ciliary hypertrichosis, and periorbital hyperpigmentation at Week 24 compared with baseline, based on institutional assessment. FLEB and ciliary hypertrichosis were assessed visually using facial photographs obtained at every study visit. Upper eyelid ptosis severity was assessed using Margin Reflex Distance 1 (MRD1), defined as the vertical distance from the central corneal light reflex to the upper eyelid margin, and Margin Reflex Distance 2 (MRD2), defined as the vertical distance from the central corneal light reflex to the lower eyelid margin. To account for inter-individual variability in eyelid anatomy, MRD2 was used as an individualized anatomical reference. Upper eyelid ptosis severity was determined based on the difference between MRD1 and MRD2 within the same eye, calculated as MRD2 − MRD1. Upper eyelid ptosis was graded as mild when the difference was less than 2 mm, moderate when the difference was 2 mm to less than 4 mm, and severe when the difference was 4 mm or more. Periorbital hyperpigmentation was evaluated by comparison with the surrounding facial skin and graded on a 5-point scale from 0–4: grade 0 = skin color comparable with other facial skin areas; grade 1 = faint pigmentation of the infraorbital fold; grade 2 = more pronounced pigmentation; grade 3 = deep dark pigmentation involving all four eyelids; and grade 4 = grade 3+ pigmentation spreading beyond the infraorbital fold.

Improvement was defined as a decrease of at least one grade or a change from “Yes” to “No” relative to baseline. Patients with a baseline grade of 0 or categorized as “No/Mild” were excluded from the improvement analysis, as further improvement could not be assessed.

IOP was measured at every visit (Screening, Baseline, Week 6, Week 12, Week 18, and Week 24) using a Goldmann applanation tonometer. If the difference between two measurements was ≤2 mm Hg, the average value was used. If the difference between two measurements was >2 mm Hg, a third measurement was taken, and the median value was used. The response rate of IOP reduction at Week 24 was defined as the proportion of patients achieving an IOP reduction of ≥15% and ≥20% from baseline.

Independent assessments were conducted for DUES, FLEB, and periorbital hyperpigmentation. Three independent evaluators reviewed facial photographs obtained at each visit and classified changes from baseline as “improved” or “no change”. PAP was considered improved if at least one of the three assessed manifestations was classified as “improved”. Ciliary hypertrichosis and upper eyelid ptosis were excluded from the independent assessment because their evaluation was considered unreliable based solely on photographic documentation. Discrepancies among evaluators were resolved through consensus during a separate online review meeting. Investigators responsible for patient care did not participate in the independent assessment.

For the subjective evaluation of changes in PAP, patients were asked to assess the overall change in their PAP symptoms based on their own perception. The evaluation was recorded as either “improved” or “no change”.

Safety was assessed by the occurrence of AEs after the administration of the investigational product during the observation period. In addition, optical coherence tomography (OCT) was performed as part of safety assessments to evaluate macular edema and cystoid macular edema (CME). OCT assessments were conducted at baseline, Week 12, and Week 24. Macular edema and CME were assessed according to predefined, protocol-specified criteria.

### 4.4. Statistical Analysis

Sample size estimation was based on a precision approach. The expected improvement rate was assumed to be 50%, based on a previous prospective study [38], in which 6 of 12 patients showed improvement in DUES at Week 24. Assuming an expected improvement rate of 50%, a two-sided significance level of 5% and a relatively small margin of error of 10.65%, the required sample size was calculated to be 85 patients. Allowing for a 20% dropout rate, the final required sample size was approximately 107 patients.

All statistical analyses were performed using SAS version 9.4 (SAS Institute, Cary, NC, USA). Descriptive statistics were used to summarize the data. Continuous variables are summarized as means and standard deviations (SDs), whereas categorical variables are presented as frequencies and percentages. Statistical significance was defined as a two-sided *p*-value of <0.05. Two-sided 95% confidence intervals (CIs) were reported where appropriate.

The mean difference in IOP between baseline and Week 24 was estimated, and a two-sided 95% CI was reported. Additionally, a paired *t*-test was conducted to compare IOP between baseline and Week 24. The IOP reduction response rate at Week 24 was presented as the proportion of patients achieving an IOP reduction of ≥15% and ≥20% from baseline.

## 5. Conclusions

This prospective, multicenter study demonstrated PAP manifestations improved after transition from PGA therapy to omidenepag isopropyl across investigator, independent, and patient-reported assessments while maintaining effective IOP control and favorable tolerability. Given the potential impact of PAP on long-term glaucoma management, these findings support treatment transition to omidenepag isopropyl as a clinically feasible management strategy for glaucoma and glaucoma suspect patients with PAP.

## Figures and Tables

**Table 1:** Patient demographics and baseline characteristics (*n* = 96).

Characteristic	Value
Sex, *n* (%)	
Male	41 (42.71%)
Female	55 (57.29%)
Age (years), mean (SD)	63.17 ± 10.46
Baseline IOP (mmHg)	16.71 ± 2.86
Diagnosis, *n* (%)	
POAG	13 (13.54%)
NTG	43 (44.79%)
Glaucoma Suspect	40 (41.67%)
PGA therapy before switching to OMDI, *n* (%)	
Bimatoprost	20 (20.83%)
Latanoprost	38 (39.58%)
Tafluprost	20 (20.83%)
Travoprost	18 (18.75%)

IOP, intraocular pressure; NTG, normal tension glaucoma; OMDI, omidenepag isopropyl; PGA, prostaglandin analog; POAG, primary open-angle glaucoma; SD, standard deviation.

**Table 2:** Baseline PAP characteristics (*n* = 96).

PAP Sign	Category	*n* (%)
DUES	Grade 0	9 (9.38%)
	Grade 1	24 (25.00%)
	Grade 2	28 (29.17%)
	Grade 3	23 (23.96%)
	Grade 4	12 (12.50%)
FLEB	No	40 (41.67%)
	Yes	56 (58.33%)
Upper eyelid ptosis	Mild	39 (40.63%)
	Moderate	44 (45.83%)
	Severe	13 (13.54%)
Ciliary hypertrichosis	No	23 (23.96%)
	Yes	73 (76.04%)
Periorbital hyperpigmentation	Grade 0	3 (3.13%)
	Grade 1	27 (28.13%)
	Grade 2	42 (43.75%)
	Grade 3	20 (20.83%)
	Grade 4	4 (4.17%)

DUES, deepening of the upper eyelid sulcus; FLEB, flattening of lower eyelid bag; PAP, prostaglandin-associated periorbitopathy.

**Table 3:** Summary of the proportion of improvement in five PAP signs at Week 24 compared with baseline.

PAP Signs	*n* (%)
DUES (*n* = 87)	
Improved *	68 (78.16)
Not Improved	19 (21.84)
FLEB (*n* = 56)	
Improved	17 (30.36)
Not Improved	39 (69.64)
Upper eyelid ptosis (*n* = 57)	
Improved	25 (43.86)
Not Improved	32 (56.14)
Ciliary hypertrichosis (*n* = 73)	
Improved	36 (49.32)
Not Improved	37 (50.68)
Periorbital hyperpigmentation (*n* = 93)	
Improved	70 (75.27)
Not Improved	23 (24.74)

* Patients were classified as improved if DUES, periorbital hyperpigmentation, or upper eyelid ptosis decreased by at least one grade/severity scale from baseline, or if FLEB or ciliary hypertrichosis changed from Yes at baseline to No at Week 24. All other patients were classified as not improved. DUES, deepening of the upper eyelid sulcus; FLEB, flattening of lower eyelid bag; PAP, prostaglandin-associated periorbitopathy.

**Table 4:** AEs reported in patients during treatment with omidenepag isopropyl 0.002% ophthalmic solution.

**Type of Event**	**Patients (*n* = 107),** ***n* (%)**
AEs	22 (20.56)
SAEs	0 (0.0)
ADR *	14 (13.08)
**Adverse Event**	**Cases (*n* = 33),** ***n* (%)**
Ocular hyperemia	5 (15.15)
Eye pain	3 (9.09)
Visual acuity reduced	3 (9.09)
Eye swelling	2 (6.06)
Vitreous floaters	2 (6.06)
Eye irritation	1 (3.03)
Eye pruritus	1 (3.03)
Eczema eyelids	1 (3.03)
Myopia	1 (3.03)
Photophobia	1 (3.03)
Blurred vision	1 (3.03)
Visual impairment	1 (3.03)
Conjunctival hyperemia	1 (3.03)
Palpitations	1 (3.03)
Chronic gastritis	1 (3.03)
Hepatic calcification	1 (3.03)
Hepatic haematoma	1 (3.03)
Liver hernia	1 (3.03)
Benign neoplasm	1 (3.03)
Shoulder fracture	1 (3.03)
Neuropathy peripheral	1 (3.03)
Paraesthesia	1 (3.03)
Oropharyngeal pain	1 (3.03)

* The adverse events assessed as “certain”, “probable”, “possible”, “conditional/unclassified” or “unassessable/unclassifiable” in terms of causality were considered ADRs that could not be ruled out. AE, adverse event; ADR, adverse drug reaction; SAE, serious adverse event.

## Data Availability

The original contributions presented in this study are included in the article/Supplementary Materials. Further inquiries can be directed to the corresponding author.

## Supplementary Materials

The following supporting information can be downloaded at: https://www.mdpi.com/article/10.3390/ph19101636/s1, Table S1: Comparison of IOP at Screening, Baseline, and Week 24 (*n* = 96); Table S2: Summary of the proportion of improvement in five PAP signs at each visit compared with baseline.

## Abbreviations

The following abbreviations are used in this manuscript:

ADR	Adverse drug reaction
AE	Adverse event
CI	Confidence interval
DUES	Deepening of upper eyelid sulcus
EP2	Prostaglandin E2 receptor
FLEB	Flattening of lower eyelid bag
FP	Prostaglandin F receptor
IOP	Intraocular pressure
MRD	Margin reflex distance
NTG	Normal-tension glaucoma
OHT	Ocular hypertension
OMDI	Omidenepag isopropyl
PAP	Prostaglandin-associated periorbitopathy
PGF2α	Prostaglandin F2-alpha
PGAs	Prostaglandin analogs
POAG	Primary open-angle glaucoma
RWE	Real-world evidence
SAE	Serious adverse event
SD	Standard deviation
