# Supplementary material for: Clinical Outcomes of Switching from Prostaglandin Analogs to Omidenepag Isopropyl in Primary Open-Angle Glaucoma and Glaucoma Suspect Patients with Prostaglandin-Associated Periorbitopathy: A Prospective, Multicenter Study
Source: Pharmaceuticals (Basel). 2026 Oct 16;19(10):1636. doi: 10.3390/ph19101636 (PMC13708616; doi:10.3390/ph19101636)
Supplement: Supplementary file 1 [file pharmaceuticals-19-01636-s001.zip › pharmaceuticals-4604377-supplementary.pdf]

Supplementary Table S1. Comparison of IOP at Screening, Baseline, and Week 24 (n = 96).

IOP (mmHg)		
**Visit**	**Mean**	**SD**
Screening*	14.56	3.0
Baseline**	16.71	2.86
Week 24***	14.57	2.34

* Screening, while patients were receiving their previous PGA therapy. **Baseline, after the 4-week washout period and before initiation of omidenepag isopropyl. ***Week 24, after 24 weeks of treatment with omidenepag isopropyl. IOP, intraocular pressure; SD, standard deviation; PGA, prostaglandin analog.

Supplementary Table S2. Summary of the proportion of improvement in five PAP signs at each visit compared with baseline.

Improvement (%)					
**Visit**	**DUES (n = 87)**	**FLEB (n = 56)**	**Upper Eyelid Ptosis (n = 57)**	**Ciliary Hypertrichosis (n = 73)**	**Periorbital Hyperpigmentation (n = 93)**
Week 6	40.23	17.86	31.58	15.07	50.54
Week 12	57.47	21.43	33.33	27.4	66.67
Week 18	66.67	32.14	43.86	43.84	72.04

DUES, deepening of the upper eyelid sulcus; FLEB, flattening of lower eyelid bag; PAP, prostaglandin-associated periorbitopathy.
